# Multifactorial Comparative Analysis of Platelet-Rich Plasma and Serum Prepared Using a Commercially Available Centrifugation Kit

**DOI:** 10.7759/cureus.48918

**Published:** 2023-11-16

**Authors:** Takayuki Okumo, Atsushi Sato, Kanako Izukashi, Masataka Ohta, Jun Oike, Saki Yagura, Naoki Okuma, Takayuki Koya, Masataka Sunagawa, Koji Kanzaki

**Affiliations:** 1 Department of Orthopedic Surgery, Showa University Fujigaoka Hospital, Yokohama, JPN; 2 Department of Physiology, Showa University Graduate School of Medicine, Tokyo, JPN; 3 Department of Orthopedic Surgery, Showa University Koto Toyosu Hospital, Tokyo, JPN

**Keywords:** vascular endothelial growth factor (vegf), leukocyte-rich prp, il-1ra, luminex assay, osteoarthritis (oa), platelet-rich plasma (prp)

## Abstract

Background

Platelet-rich plasma (PRP) is an autologous product prepared by centrifuging whole blood. PRP is reported to have high tissue repair potential and anti-inflammatory properties. Recently, PRP has become a potential treatment option for osteoarthritis, contributing to pain relief and locomotive improvement. However, the underlying therapeutic mechanisms and key biochemical factors in PRP remain unclear. This study aimed to estimate the major factors for tissue repair involved in PRP treatment by comparing between serum and PRP prepared from the same patients using the Luminex assay.

Methodology

Blood samples were collected from nine healthy volunteers, and serum and PRP were prepared. PRP was prepared using a PEAK^©︎^ PRP SYSTEM kit of DePuy Synthes Mitek Sports Medicine (Raynham, Massachusetts, USA), which is a commercially available PRP preparation kit. The white blood cell count, hemoglobin level, and platelet count were automatically measured for both whole blood and PRP in the hospital’s clinical laboratory using the XE-5000™ Automated Hematology System (Sysmex, Kobe, Japan). Comparative analysis of biological factors was then performed using the Luminex assay on serum and PRP.

Results

PRP was found to have significantly higher white blood cell and platelet counts and lower hemoglobin levels than whole blood. Furthermore, PRP contained significantly higher levels of various factors, including interleukin (IL)-1ra, IL-10, IL-13, C-C motif chemokine ligand (CCL)-2, CCL3, CCL4, CCL8, CCL13, CCL21, C-X-C motif chemokine ligand (CXCL)-10, matrix metalloproteinase (MMP)-3, MMP-9, cluster of differentiation (CD) 40 ligand, vascular endothelial growth factor (VEGF), VEGF-C, platelet-derived growth factor (PDGF)-AB, PDGF-BB, and bone morphogenic protein (BMP)-2. Additionally, IL-1ra and IL-4 showed significant correlations with white blood cell counts in PRP, whereas VEGF had a significant correlation with platelet counts.

Conclusions

PRP contains various factors in higher quantities than serum. Specifically, the notable increase in the anti-inflammatory cytokine IL-1ra is suggested to play a key role as a major therapeutic mechanism of PRP.

## Introduction

Autologous cellular therapies that use platelet-rich plasma (PRP) have the potential to play an important role in various regenerative treatments [1]. Tissue repair strategies for treating musculoskeletal disorders and chronic recalcitrant wounds or skin ulcers are needed worldwide. Platelet-derived growth factors contained in PRP are expected to promote angiogenesis and mesenchymal stem cell proliferation and have been increasingly used in sports medicine, mainly to promote tissue repair for muscle injuries and rotator cuff tears of the shoulder [2,3]. Recently, some studies have shown the efficacy of PRP therapy in treating tendinopathies, such as patellar tendinitis and Achilles tendinitis improving pain and function, and shortening the time from injury to return to sports, raising interest in its anti-inflammatory effects [3]. Considering both the anti-inflammatory and tissue repair properties of PRP therapy, its use in treating osteoarthritis is gaining interest [4].

Osteoarthritis is a chronic degenerative disease in which patients suffer from pain and locomotive dysfunction due to joint deformity and inflammatory response [5]. Because the pathogenesis of the disease has been elucidated, it has become clear that chronic mild inflammation in the synovium and abnormal bone metabolism in the subchondral bone, including bone cysts and osteophyte formation, are involved in its pathogenesis [6]. Nonsteroidal anti-inflammatory drugs and corticosteroids are recommended as treatment strategies for this disease, along with patient education and exercise therapy as core treatment strategies [7]. Intra-articular hyaluronic acid injection is also used in outpatient practice; however, PRP has shown a better clinical outcome in a recent study comparing hyaluronic acid [8,9]. To elucidate the superior therapeutic mechanism of PRP, analyzing the biochemical factors contained in PRP may be needed, particularly considering the factors involved in the pathophysiology of osteoarthritis, which is not fully understood, although several studies have been conducted [10]. Therefore, this study aimed to determine the factors contained in PRP prepared using a commercially available centrifugation kit by comparing them with those contained in serum.

## Materials and methods

In this study, we analyzed PRP obtained from generally healthy adults. People with medical ailments, surgical history, and internal medication use, aged under 20 or over 60 years were excluded from this study. This study involved the examination of blood counts and a comparative multifactorial analysis of serum and PRP obtained from nine generally healthy adults (including six males and three females) with ages ranging from 28 to 51 years. Before blood sample collection, the purpose and protocol of the study were explained to each participant, and informed consent for the study was obtained after confirmation of their lack of medical ailments, surgical history, and internal medication use. This study was conducted according to the ethical guidelines for research involving human subjects at Showa University. The investigation was implemented after obtaining approval from Showa University (22-149-B). After obtaining informed consent and providing explanations to the research participants, blood samples were collected. The blood sample collection procedure was as follows: 2 mL of blood was collected for whole blood cell count, 8 mL for serum preparation, and 27 mL for PRP preparation. To prepare PRP, 3 mL of acid citrate dextrose solution A (ACD-A) was mixed into the collection syringe to prevent coagulation.

The blood samples were subjected to centrifugation following the protocol of the PEAK^©︎^ PRP SYSTEM kit of DePuy Synthes Mitek Sports Medicine (Raynham, Massachusetts, USA), resulting in the retrieval of 3 mL of PRP. Of the retrieved 3 mL of PRP, 1 mL was allocated for multiplex assays, 1 mL for blood cell counts, and 1 mL for storage. The 8 mL of blood collected for serum preparation was stored at room temperature for 30 minutes to allow clot formation. Subsequently, the blood sample was centrifuged at 1,000 × g for 15 minutes; then, the supernatant was collected. The PRP and serum samples for comparative multifactorial assay and storage were frozen at −80℃ until the time of analysis.

Whole blood samples for blood cell counts were placed in ethylenediaminetetraacetic acid-containing collection tubes and immediately analyzed in the facility’s testing laboratory using the XE-5000™ Automated Hematology System (Sysmex, Kobe, Japan). Blood cell counts, including white blood cell (WBC) count, hemoglobin (Hb) level, and platelet (Plt) count, were measured for both whole blood and PRP, and comparative analyses were performed.

The prepared serum and PRP were outsourced to Genetic Lab Co., Ltd. (Hokkaido, Japan) for the Luminex assay. The analysis was performed using the BioPlex-200 system from Bio-Rad Laboratories (Richmond, CA), and measurements were performed for the following parameters: interleukin (IL)-1 beta, IL-1ra, IL-4, IL-6, IL-10, IL-13, and IL-17; C-C motif chemokine ligand (CCL) 2, CCL3, CCL4, CCL8, CCL13, CCL15, and CCL21; C-X3-C motif chemokine ligand (CX3CL)-1; C-X-C motif chemokine ligand (CXCL)-8 and CXCL10; matrix metalloproteinase (MMP)-1, MMP-3, MMP-9, and MMP-13; cluster of differentiation (CD)-40 ligand and CD44; vascular endothelial growth factor (VEGF) and VEGF-C; platelet-derived growth factor (PDGF)-AB and PDGF-BB; and bone morphogenic protein (BMP)-2. Conducting a Luminex assay typically involves several key steps, and a general overview of the process is presented below.

First, the Bio-Plex 200 system was set up and calibrated according to the manufacturer’s instructions. After preparing all necessary reagents, including assay beads coated with specific antibodies, standards, and samples, the lyophilized bead mix was reconstituted with the supplied buffer or diluent, and the bead suspension was thoroughly vortexed for at least 30 seconds. Samples or standards were then added to the microplate wells. The samples were estimated to fall within the linear range of the assay, which were prepared to be diluted three times. The microplate containing the samples, standards, and bead suspension was then incubated at room temperature for two hours to allow the antigen-antibody reaction to occur. After incubation, the microplate wells were washed to remove any unbound material or debris. The detection antibody specific to the analyte of interest in each well was then added and incubated at room temperature for one hour. The microplate was washed again to remove unbound detection antibodies, and an appropriate streptavidin-phycoerythrin detection reagent was applied to each well for signal amplification and detection. For data acquisition, the prepared microplate was loaded into the Bio-Plex 200 system, and the assay was run to read the fluorescence signals from the beads in each well. The system collected data on the fluorescence intensity of each bead. Data were then analyzed using the Bio-Plex Manager software.

For statistical analysis, JMP Pro ver. 16 was used. The blood counts for whole blood and PRP and the levels of each factor in serum and PRP measured using the Luminex assay were expressed as means and standard deviations. Because this was a comparative analysis using biological samples collected from the same patients, a paired t-test was performed for each, and the significance level was set at 5%. Correlations with blood counts were analyzed for each analyte measured with the Luminex assay.

## Results

**Figure 1:**
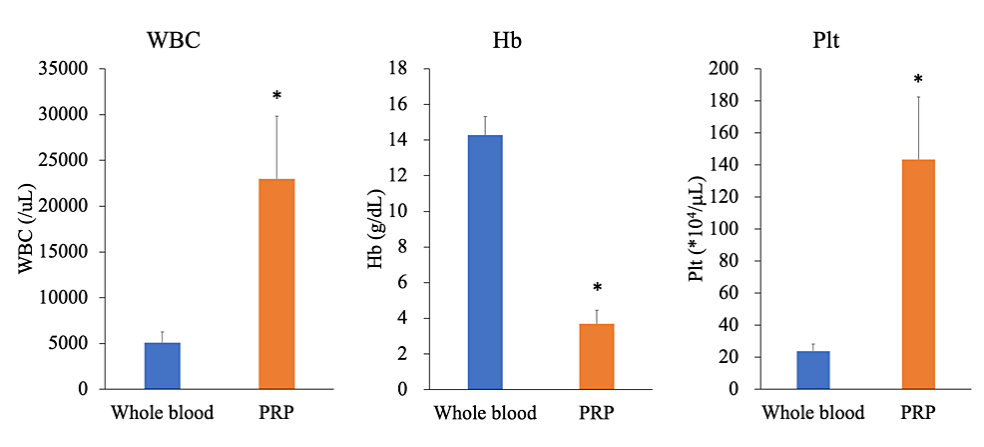
Blood cell count for whole blood and PRP. *: P < 0.05 vs. whole blood assessed by the paired t-test. WBC = white blood cell; Hb = hemoglobin; Plt = platelet; PRP = platelet-rich plasma

The WBC count was 5,116 ± 1,139/µL in whole blood and 23,006 ± 6,819/µL in PRP. The Hb level was 14.3 ± 1.0 g/dL in whole blood and 3.7 ± 0.8 g/dL in PRP. The Plt count was 23.8 ± 4.4 × 10^4^/µL in whole blood and 143.4 ± 39.1 × 10^4^/µL in PRP. PRP had significantly higher WBC count (p < 0.001), lower Hb level (p < 0.001), and higher Plt count (p* *< 0.001) (Figure 1).

**Figure 2:**
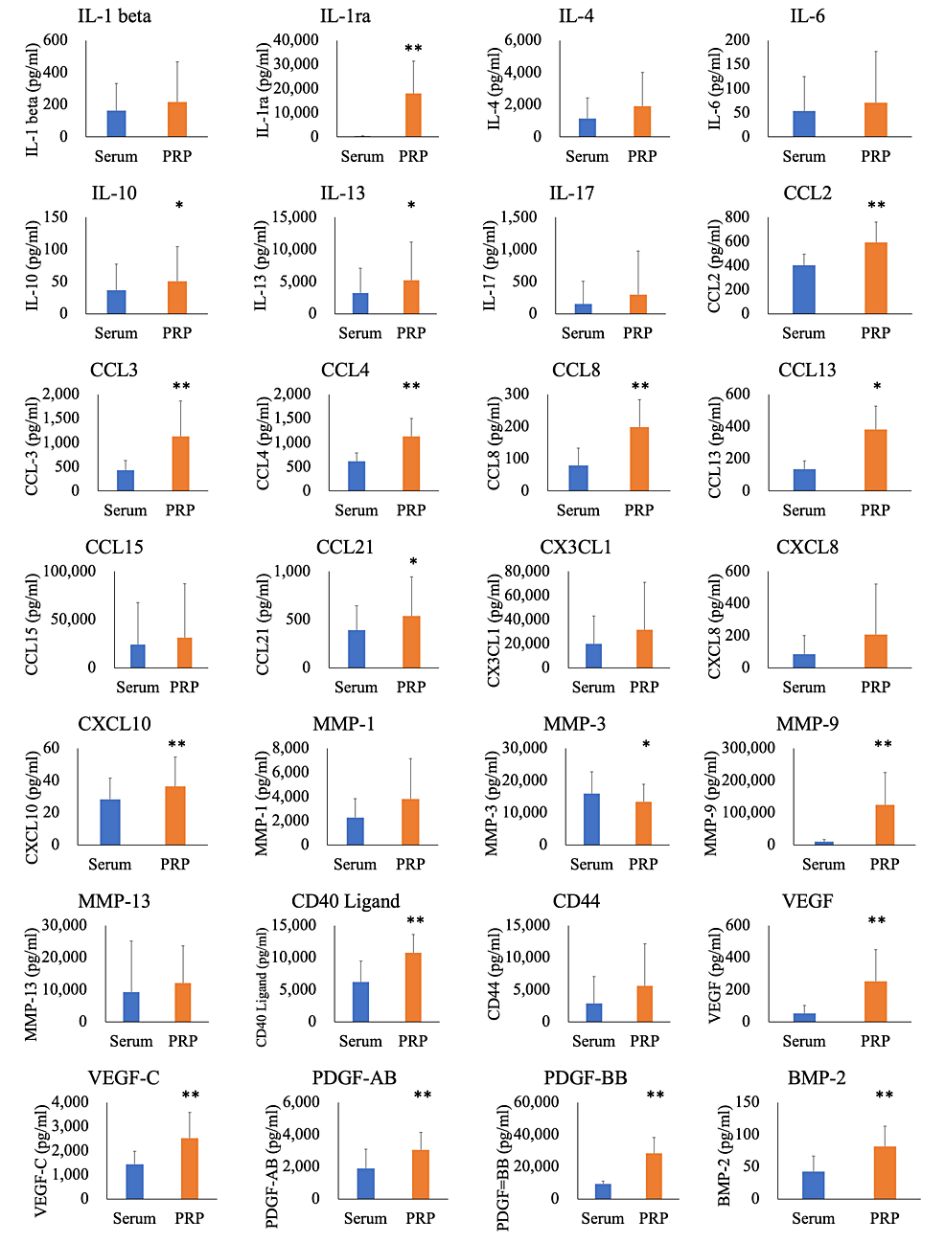
Comparison between serum and PRP using the Luminex assay. *: P < 0.05, **: P < 0.01 vs. serum assessed by the paired t-test. IL = interleukin; CCL = C-C motif chemokine ligand; CXCL = C-X-C motif chemokine ligand; CX3CL = C-X3-C motif chemokine ligand; MMP = matrix metalloproteinase; CD = cluster of differentiation; VEGF = vascular endothelial growth factor; PDGF = platelet-derived growth factor; BMP = bone morphogenic protein; PRP = platelet-rich plasma

Comparison of serum and PRP showed that various factors were significantly more abundant in the PRP group than those in the serum group (Figure 2). Specifically, the levels of IL-1ra (p = 0.004), IL-10 (p = 0.024), IL-13 (p = 0.026), CCL2 (p = 0.001), CCL3 (p = 0.005), CCL4 (p < 0.001), CCL8 (p = 0.012), CCL13 (p < 0.001), CCL21 (p = 0.045), CXCL10 (p = 0.006), MMP-3 (p = 0.041), MMP-9 (p = 0.010), CD40 ligand (p = 0.006), VEGF (p = 0.006), VEGF-C (p = 0.008), PDGF-AB (p = 0.001), PDGF-BB (p < 0.001), and BMP-2 (p < 0.001) were significantly higher in PRP than in serum.

Subsequently, a correlation analysis was performed between each analyte and the WBC count, Hb level, and Plt count in PRP (Table 1). Significant correlations were found between WBC counts and IL-1ra (correlation coefficient (CC) = 0.842, 95% confidence interval (CI) = 0.404-0.966, p = 0.004), IL-4 (CC = 0.869, 95% CI = 0.483-0.972, p = 0.002), and CCL4 (CC = 0.771, 95% CI = 0.218-0.949, p = 0.015). No factors were found to be correlated with Hb levels. VEGF (CC = 0.684, 95% CI = 0.036-0.927, p = 0.042) was significantly correlated with Plt count.

**Table 1:** Correlation analysis between biochemical factor levels of PRP and blood cell counts. IL = interleukin; CCL = C-C motif chemokine ligand; CXCL = C-X-C motif chemokine ligand; CX3CL = C-X3-C motif chemokine ligand; MMP = matrix metalloproteinase; CD = cluster of differentiation; VEGF = vascular endothelial growth factor; PDGF = platelet-derived growth factor; BMP = bone morphogenic protein; PRP = platelet-rich plasma

	WBC					Hb					Plt				
	Correlation coefficient	95% confidence interval			P-value	Correlation coefficient	95% confidence interval			P-value	Correlation coefficient	95% confidence interval			P-value
IL-1 beta	0.006	−0.660	~	0.667	0.988	0.095	−0.607	~	0.714	0.808	−0.022	−0.676	~	0.652	0.955
IL-1ra	0.842	0.404	~	0.966	0.004**	−0.059	−0.696	~	0.630	0.880	0.629	−0.059	~	0.912	0.069
IL-4	0.869	0.483	~	0.972	0.002**	−0.543	−0.887	~	0.188	0.130	0.160	−0.564	~	0.745	0.682
IL-6	−0.159	−0.744	~	0.565	0.682	−0.354	−0.824	~	0.405	0.350	−0.528	−0.882	~	0.208	0.143
IL-10	0.417	−0.341	~	0.847	0.264	−0.649	−0.917	~	0.025	0.058	−0.264	−0.789	~	0.485	0.492
IL-13	−0.011	−0.670	~	0.658	0.976	0.224	−0.517	~	0.773	0.563	0.217	−0.522	~	0.770	0.574
IL-17	0.027	−0.648	~	0.679	0.945	−0.491	−0.871	~	0.256	0.179	−0.125	−0.729	~	0.587	0.747
CCL2	−0.378	−0.833	~	0.381	0.315	−0.020	−0.675	~	0.653	0.959	−0.478	−0.866	~	0.273	0.193
CCL3	−0.404	−0.842	~	0.355	0.280	−0.012	−0.671	~	0.657	0.974	−0.192	−0.759	~	0.541	0.620
CCL4	0.771	0.218	~	0.949	0.015*	−0.304	−0.805	~	0.451	0.426	0.389	−0.370	~	0.837	0.300
CCL8	0.024	−0.692	~	0.717	0.954	0.625	−0.141	~	0.923	0.097	0.457	−0.364	~	0.879	0.255
CCL13	0.613	−0.086	~	0.908	0.079	−0.178	−0.75	~	0.551	0.646	0.193	−0.540	~	0.760	0.620
CCL15	−0.205	−0.765	~	0.531	0.595	0.286	−0.466	~	0.799	0.455	−0.013	−0.671	~	0.656	0.972
CCL21	0.480	−0.270	~	0.868	0.191	−0.500	−0.873	~	0.246	0.170	−0.043	−0.687	~	0.639	0.912
CX3CL1	−0.291	−0.800	~	0.462	0.447	0.352	−0.406	~	0.824	0.352	0.051	−0.634	~	0.692	0.897
CX3CL8	0.618	−0.079	~	0.909	0.076	−0.617	−0.908	~	0.080	0.077	−0.126	−0.729	~	0.587	0.745
CXCL10	−0.433	−0.852	~	0.324	0.244	0.205	−0.531	~	0.765	0.597	−0.421	−0.848	~	0.337	0.258
MMP-1	−0.280	−0.796	~	0.471	0.465	0.184	−0.546	~	0.756	0.635	0.321	−0.436	~	0.812	0.400
MMP-3	0.352	−0.407	~	0.824	0.352	−0.390	−0.837	~	0.370	0.299	−0.027	−0.679	~	0.648	0.943
MMP-9	0.617	−0.079	~	0.909	0.077	0.061	−0.628	~	0.697	0.876	0.525	−0.213	~	0.882	0.147
MMP-13	0.075	−0.620	~	0.704	0.847	−0.457	−0.860	~	0.297	0.216	−0.047	−0.690	~	0.637	0.903
CD40 ligand	0.270	−0.480	~	0.792	0.483	−0.051	−0.691	~	0.634	0.895	0.525	−0.213	~	0.882	0.147
CD44	0.377	−0.383	~	0.833	0.317	0.360	−0.399	~	0.827	0.341	0.522	−0.218	~	0.881	0.150
VEGF	0.197	−0.537	~	0.761	0.612	0.435	−0.322	~	0.853	0.242	0.684	0.036	~	0.927	0.042*
VEGF-C	−0.186	−0.756	~	0.545	0.632	0.284	−0.468	~	0.798	0.459	0.192	−0.541	~	0.759	0.621
PDGF-AB	0.218	−0.521	~	0.771	0.573	0.072	−0.621	~	0.703	0.854	0.100	−0.604	~	0.716	0.799
PDGF-BB	0.161	−0.563	~	0.746	0.679	0.297	−0.457	~	0.803	0.438	0.607	−0.095	~	0.906	0.083
BMP-2	0.194	−0.539	~	0.760	0.616	−0.404	−0.842	~	0.355	0.280	−0.436	−0.853	~	0.320	0.240

## Discussion

PRP is an autologous product prepared by centrifuging whole blood, and it is reported to have high tissue repair potential and anti-inflammatory properties [1]. Recently, PRP treatment has been considered a therapeutic strategy for osteoarthritis, contributing to pain relief and locomotive improvement [3,4].

PRP preparations are categorized mainly into two types, namely, leukocyte-rich PRP (LR-PRP) preparations, having more WBCs than baseline, and leukocyte-poor PRP (LP-PRP) preparations, having less than baseline [11]. Recently, whether LR-PRP provides a superior therapeutic benefit over LP-PRP has been debated. However, several randomized controlled trials have found no significant differences in clinical outcomes between the two PRP types [12,13]. Furthermore, a study comparing placebo (saline), LR-PRP, and LP-PRP in treating patellar tendinopathy also failed to show significant differences in clinical outcomes [14], casting skepticism on the efficacy of PRP therapy itself. In contrast, it has been reported that LR-PRP resulted in significant histological improvements in a rabbit Achilles tendonitis model compared with LP-PRP [15].

In this study, LR-PRP was prepared using a commercially available centrifugation kit. To assess the blood count in PRP, whole blood collected from the same subjects was used as a control. PRP exhibited significantly higher WBC and Plt count than whole blood. It has been reported that for PRP treatment to be effective in treating osteoarthritis, the Plt count in PRP should be at least 10 billion/8 mL (1.25 billion/mL) [16]. In this study, the Plt count was approximately 1.43 billion/mL, suggesting the potential for favorable clinical outcomes. In contrast, Hb levels were significantly lower. Additionally, because Hb has been reported to be responsible for exacerbating synovitis, which is crucial to the development of osteoarthritis [17], PRP with lower Hb levels is desirable, and the agents prepared in this study revealed a lower level.

It has also become evident that the concentrations of IL-1ra and VEGF were notably elevated in PRP compared with those in serum. Furthermore, these two factors showed significant positive correlations with WBC and Plt count, respectively. IL-1ra is an anti-inflammatory cytokine that antagonizes IL-1 alpha and IL-1 beta signaling and exists in two subtypes: a 17-kDa form secreted by monocytes, neutrophils, and macrophages, and an 18-kDa form that remains in the cytoplasm of epithelial cells, endothelial cells, and fibroblasts [18,19]. In this study, IL-1ra showed a significant positive correlation with WBC count, suggesting the superiority of LR-PRP. In contrast, according to previous reports, the average concentration of IL-1ra in LP-PRP was lower than that in this study [20], indicating that using LR-PRP may be more advantageous due to its enhanced anti-inflammatory effects mediated by IL-1ra. At present, IL-1ra is not currently in clinical use, and only a pilot study has been reported to assess its safety and efficacy in patients with anterior cruciate ligament injuries [21]. VEGF plays an important role in tissue repair by promoting endothelial cell proliferation and tissue vascularization and stimulating the accumulation of stem and immune cells necessary for tissue repair in inflamed and injured tissues [22]. In this study, VEGF showed a significant positive correlation with the Plt count, consistent with previous findings [23,24]. VEGF has been reported to promote tissue repair in meniscus injuries [25] but has also been associated with degenerative changes in articular cartilage [26,27], raising concerns about its contribution to osteoarthritis progression. Another important aspect to consider is the effect of PRP on synovial cells. It has been reported that PRP promotes the secretion of MMPs, which are enzymes involved in the degradation of articular cartilage [28].

PRP comprises a lot of components that may have various effects on anabolic and catabolic changes in tissues. It has generally shown positive clinical results, including pain relief and improved locomotive function in patients with osteoarthritis [4,8,9]. However, long-term studies are required to clarify its potential to inhibit disease progression. Based on the results of this study, which highlighted IL-1ra as an important anti-inflammatory factor, further investigation of the therapeutic effects of LR-PRP is needed.

This study has several limitations. First, the participants were healthy adults; therefore, further analysis of PRP in patients with osteoarthritis is required. However, the validity of this study is to investigate the influence of preparing PRP from blood samples affecting the level of biochemical mediators contained in PRP, so it is necessary to remove as many possible biases as we can, such as past medical history, current ailment, surgical history, and medications. Therefore, we believe it is applicable that only healthy adults were included in this study. Second, the number of subjects was relatively small. Although no outliers were observed in any parameter, a larger sample size would have strengthened the validity of the findings. However, a previous study has shown that PRP has much IL-1ra [20], which is relevant to our study, so it may be appropriate to compare PRP and serum originating from the same subject using a paired t-test. Finally, this study only focused on LR-PRP prepared using only one preparation kit, and a similar analysis should be performed for LP-PRP.

## Conclusions

In this study, a multifactorial analysis using the Luminex assay was performed on serum and PRP prepared from blood samples of the same subject and PRP contained a larger amount of various kinds of biochemical mediators than serum. Furthermore, IL-1ra and VEGF were significantly more concentrated and correlated with leukocyte and platelet counts, respectively. In light of previous reports on these two biological factors, LR-PRP prepared using the PEAK PRP SYSTEM may have favorable clinical results. In particular, the significant increase in IL-1ra, about 40 times higher than serum, suggests that it plays an important role in PRP treatment, and the anti-inflammatory effect may shed light on the therapeutic mechanism of PRP. However, the results of this study are not directly applicable to clinical practice because the study was performed on a relatively small number of subjects, especially healthy adults without any ailments in medical history. Thus, to determine if PRP is a potential therapeutic strategy for osteoarthritis or other degenerative diseases, it is necessary to examine whether PRP made from patients with the disease has similar biochemical factor profiles to those of healthy adults. Therefore, further studies are needed to elucidate the efficacy of PRP treatment for osteoarthritis patients, such as multifactorial investigation for PRP in osteoarthritis patients.
